# Arrhythmias following Revascularization Procedures in the Course of Acute Myocardial Infarction: Are They Indicators of Reperfusion or Ongoing Ischemia?

**DOI:** 10.1155/2013/160380

**Published:** 2013-01-31

**Authors:** Ersan Tatli, Güray Alicik, Ali Buturak, Mustafa Yilmaztepe, Meryem Aktoz

**Affiliations:** ^1^Department of Cardiology, Trakya University Hospital, Edirne, Turkey; ^2^Department of Cardiology, Ada Tıp Hospital, Sakarya, Turkey; ^3^Cardiology Department, Acibadem University School of Medicine, Istanbul, Turkey

## Abstract

*Objective*. The most important step in the treatment of ST elevation myocardial infarction is to sustain myocardial blood supply as soon as possible. The two main treatment methods used today to provide myocardial reperfusion are thrombolytic therapy and percutaneous coronary intervention. In our study, reperfusion arrhythmias were investigated as if they are indicators of coronary artery patency or ongoing ischemia after revascularization. *Methods*. 151 patients with a diagnosis of acute ST elevation myocardial infarction were investigated. 54 patients underwent primary percutaneous coronary intervention and 97 patients were treated with thrombolytic therapy. The frequency of reperfusion arrythmias following revascularization procedures in the first 48 hours after admission was examined. The relation between reperfusion arrhythmias, ST segment regression, coronary artery patency, and infarct related artery documented by angiography were analyzed. *Results*. There was no statistically significant difference between the two groups in the frequency of reperfusion arrhythmias (*P* = 0.355). Although angiographic vessel patency was higher in patients undergoing percutaneous coronary intervention, there was no significant difference between the patency rates of each group with and without reperfusion arrythmias. *Conclusion*. Our study suggests that recorded arrhythmias following different revascularization procedures in acute ST elevation myocardial infarction may not always indicate vessel patency and reperfusion. Ongoing vascular occlusion and ischemia may lead to various arrhythmias which may not be distinguished from reperfusion arrhythmias.

## 1. Introduction

The term, reperfusion arrhythmias, was used in the first studies of thrombolytic therapy guided revascularization in acute myocardial infarction. To confirm the presence of vessel patency and successful reperfusion of the myocardiumfollowing thrombolytic therapy, electrocardiographic data in addition to clinical and laboratory measures are used by the clinicians. These include normalization or more than 50% regression of ST elevation, T-wave inversion, and any other arrythmia observed in electrocardiography. The most frequently observed arrhytmias that are defined as reperfusion arrhytmias are ventricular premature contractions, sustained or nonsustained episodes of ventricular tachycardia, accelerated idioventricular rhythm, atrial fibrillation, and ventricular fibrillation. These arrhythmias are thought to be indicators of successful reperfusion. However, in some studies it was mentioned that these arrhytmias may be due to ongoing myocardial cell damage and ischemia [1].

In acute myocardial infarction, ventricular tachycardia (VT) and ventricular fibrillation (VF) may occur due to either complete occlusion or reperfusion. Rhythm disorders associated with coronary occlusion are defined as ischemic arrhytmias, whereas arrhythmias occurring as the result of increased myocardial perfusion are called reperfusion arrhythmias [2]. Accelerated idioventricular rhythm may be a marker of early reperfusion and continuing arterial patency. In a previous study, the presence of accelerated idioventricular rhytm combined with normalization of ST segments was demonstrated to indicate successful reperfusion in patients treated with thrombolytics and there was no requirement for emergency coronary angiography and rescue percutaneous coronary intervention (PCI) in this group of patients [3].

The aim of our study is to observe the arrhythmias after application of different revascularization treatment modalities (thrombolysis or primary) and investigate whether these are indicators of arterial patency or ischemia due to ongoing vascular occlusion by reviewing the arrhythmias recorded in the first 48 hours. In addition, reperfusion arrhythmias observed after thrombolysis and percutaneous revascularization were compared in this study.

## 2. Material and Methods

### 2.1. Patient Groups and Clinical Features

The data of patients treated by either thrombolysis or percutaneous coronary intervention with a diagnosis of acute ST elevation myocardial infarction followed in our clinic between the years 2008 and 2010 were evaluated retrospectively in this study. Patients with non-ST elevation myocardial infarction, unstable angina pectoris, cardiac conduction system disorders, permanent pacemakers, mechanical ventilation support, cardiogenic shock, and survivors of cardiac arrest due to acute myocardial infarction were excluded from the study group in addition to the individuals to whom rescue PCI was performed following the first 24 hours of thrombolytic therapy. The diagnosis of STEMI was based on recently published criteria of the European guidelines [4]. Time of diagnosis, patient age, gender, height, weight, hypertension, diabetes mellitus, history of previous MI, smoking, ECG, lipid profile, urea, creatinine, blood glucose, troponin I, and potassium values were recorded. The patients' blood samples were analyzed by Siemens ADVIA 1800 autoanalyzer (Siemens Healthcare Diagnostics, Tarrytown NY). Same brand of kits were used for each patient. 

Thrombolytic therapy was applied to 97 patients within 12 hours after the onset of chest pain. Coronary angiography was performed at a median of 112 minutes after the initiation of thrombolytic therapy. We did not give thrombolytic therapy to patients after 12 hours after the onset of symptoms because no mortality benefit has been demonstrated. 

### 2.2. Electrocardiographic and Echocardiographic Data

GEM ECG-9020 Cardiofax Nihon Kohden (Tokyo, Japan) system was used to obtain a twelve lead electrocardiography. Infarct localizations were classified according to the place of ST segment elevations in ECG derivations as anterior, inferior, right ventricular inferior, and lateral MI. After the treatment, ST segment regression ratio was defined in derivations with ST segment elevation. ST segment regression ratio was classified as over 50% and less than 50%. Echocardiographic examination was performed by Vivid 7 (General Electric Medical Systems, Milwaukee, WI, USA). 

### 2.3. Cardiac Catheterization, Coronary Intervention, and Assessment of Coronary Flow

Coronary angiography and percutaneous coronary interventions were performed with Philips Integris H 3000 (Eindhoven, The Netherlands). Coronary angiography was performed via the femoral artery; 4000 units of heparin and an intracoronary bolus of 100 *μ*g glyceryl trinitrate were given to all patients before angiography. Infarct related arteries were classified as LAD (left anterior descending artery), CX (circumflexartery), proximal RCA (right coronary artery), and distal RCA. After the diagnostic angiography, PCI was performed in the usual manner with balloon catheters with or without stenting. This was performed after the administration of additional heparin to maintain the activated clotting time at >300 seconds. After the PCI, heparin was continuously infused to the patients for 48 hours and was adjusted to maintain the activated clotting time at >180 seconds. The two groups received acetyl salicylic acid and clopidogrel. Patients received conventional drug therapy based on individual needs. Beta-blocker therapy (metoprolol) was initiated to patients without contraindications in the early course of acute myocardial infarction in the two groups. After the treatment the patency of the infarct related artery was determined by evaluating TIMI flow ratesof coronary angiography images [5].

### 2.4. ECG Monitorization and Definitions

Arrhythmias, recorded in the first 48 hours by Nihon Kohden Brand ECG monitoring system (BSM-5105 Tokyo-Japan), were evaluated. Whether the reperfusion arrhythmias have occurred or not was determined by examining each patient's records individually. Frequent Ventricular Premature Beat (VPB) was defined as more than 5 VPBs per minute. Accelerated Idioventricular Rhythm (AIVR) was defined as a ventricular rhythm with a rate of 60 to 125 beats/minute and frequent episodes of “slow ventricular tachycardia.” Nonsustained ventricular tachycardia (VT) was defined as 3 or more consecutive ventricular ectopic beats at a rate >100 beats/minute and lasting <30 seconds. Sustained VT was defined as the last longer than 30 seconds or cause hemodynamic compromise that requires intervention. Ventricular fibrillation was recognized by the presence of irregular ondulations of varying contour and amplitude. The conduction blocks at the atrioventricular (AV) node or intraventricular conduction systems were assessed.

### 2.5. Statistical Analysis

NCSS (Number Cruncher Statistical System) Statistical Software 2007 & PASS 2008 (Utah, USA) program was used for statistical analysis (NCSS 2007 serial number: N7H5-J8E5-D4G2-H5L6-W2R7). Descriptive statistical methods were used for evaluating data from the study groups (mean, standard deviation). For the comparison of two groups, data showing normal distribution were compared by student's *t*-test and data not displaying normal distribution were compared by Mann-Whitney *U*-test. For the comparison of qualitative data, chi-square test and Fisher's exact chi-square tests were used. *P* < 0.05 was considered statistically significant.

## 3. Results

151 patients with STEMI were included in the study. To 54 (35.8%) cases primary PCI was performed, and 97 (64.2%) cases were given thrombolytic drug therapy. The age of patients ranged between 26 and 88 years. Mean age was 58.56 ± 12.80 years. 118 patients (78.1%) were male and 33 (21.9%) were female (Table 1). There was not any statistically significant difference between primary PCI and thrombolytic treatment groups in terms of age, height, weight, and body mass index. And also mean levels of demographic data were not significantly different between the men and women (*P* > 0.05). There was no statistically significant difference between the two groups in hypertension, diabetes mellitus, smoking, history of coronary artery disease, preinfarction angina, and previous history of MI (*P* > 0.05). Systolic blood pressures, inhospital cardiovascular events, ejection fractions, onset of chest pain, and time to implementation of treatment (pain-reperfusion period) did not show a significant difference between the two groups (*P* > 0.05). The time from onset of pain to treatment time in the primary PCI and thrombolytic groups was, respectively, 4.25 ± 3.83 hours and 3.35 ± 1.87 hours (*P* = 0.317). Tables 1 and 2 show demographic and clinical characteristics of both groups.

Statistically significant differences were not found in terms of the incidence of reperfusion arrhythmias between two treatment modalities (*P* < 0.05). In the primary PCI group, 45 patients (83.3%) displayed reperfusion, while in 86 patients (88.7%) of the trombolytic group, reperfusion arrhytmias were observed (*P* = 0.355). When we evaluate the incidence of reperfusion arrhytmias separately, the rate of AIVR in the thrombolytic group was significantly higher than the PCI group: 71 patients (73.2%) and 27 patients (50%), respectively, with a *P* < 0.05 (Table 3). The rate of atrial fibrillation was significantly higher in thrombolytic treatment group compared to primary PCI group: 26 patients (26.8%) and 7 patients (13%), respectively, (*P* < 0.05). No statistically significant difference between the two groups in terms of sustained ventricular tachycardia, nonsustained ventricular tachycardia, ventricular fibrillation, frequent ventricular premature beats, and atrioventricular block was found (*P* > 0.05). ST segment regression more than 50% after revascularization was found significantly higher in primary PCI group compared with thrombolytic therapy group (Table 4). TIMI flow was assessed in both groups and no difference was found according to the type of treatment. The association of ST segment regression and reperfusion arrythmias was compared between the two groups. A statistically significant relationship was found in the thrombolytic therapy group (*P* < 0.01) compared with the primary PCI group (*P* > 0.05). The patients with more than 50% ST segment regression who received thrombolytic therapy exhibited significantly higher rates of reperfusion arrhythmias (Table 5). In addition, TIMI flow rates and reperfusion arrhythmias were examined (Table 6). There was no relationship between TIMI flow and reperfusion arrhythmias in primary PCI group (*P* > 0.05). On the contrary, reperfusion arrhythmia frequency was significantly lower for the patients with TIMI 1 flow in thrombolytic threapy group (*P* < 0.05). There was no statistically significant relationship between TIMI 0, TIMI 2, TIMI 3 flow rates and reperfusion arrhythmias (*P* > 0.05). Interestingly, the frequency of reperfusion arrhythmias was higher in the patients whose infarct related artery was left anterior descending artery (LAD) in primary PCI group (*P* < 0.05, Table 7).

## 4. Discussion

In our study, the incidence of reperfusion arrhythmias detected in the first 48 hours did not differ significantly between the two treatment groups. 83.3% of patients in primary PCI group and 88.7% of the thrombolytic group, had at least one reperfusion arrhythmia. When the rates of different arrhythmia findings were examined separately, the ratio of sustained VT, nonsustained VT, VF, frequently VEA, and AV block was similar between the two groups. However AIVR and AF ratio were higher in the thrombolytic group.

The incidence of the development of AF in acute MI is about 5–10% and it is known that AF development in acute MI is usually due to impaired left ventricular function or poor reperfusion [5]. Celik et al. reported that in patients who underwent PTCA, p dispersion was reduced, showing that successful reperfusion may reduce the likelihood of development of AF [6]. On the contrary, the incidence of AF could increase in patients with poor reperfusion. The left ventricular functions in both groups of patients were normal in this study. Therefore, the cause of AF was thought to be residuel ischemia or poor reperfusion instead of impaired left ventricular functions. Unlike thrombolytic group, we observed significantly lower rates of AF in PTCA group. Six et al. investigated the predictive value of ventricular arryhtmias as an indicator of angiographic arterial patency after thrombolytic therapy. Among these, AIVR was the most sensitive and specific arrythmia in cases of successful reperfusion [7]. In contrast, Bonnemeier et al. demonstrated that only 19 of 125 patients who were successfully treated with primary PCI exhibited AIVR which indicated a poor relationship between TIMI 2 or 3 flow and reperfusion arrhythmia and declared that AIVR may not be used as a reperfusion criteria [8].

Terkelsen et al. mentioned that although AIVR does not meet criteria for reperfusion, it may be an indicator of more extensive myocardial damage and delayed microvascular reperfusion in a study of 503 patients who were treated by primary PTCA [9]. In another study, Gibson et al. [10] exhibited the development of VT and VF in 3491 patients with STEMI after thrombolytic therapy, and reported that cases who developed VT or VF had TIMI 0–2 flow.

The relationship between arterial patency rates demonstrated via coronary angiography (TIMI flow grades). The analysis of TIMI flows allows better understanding of the relationship between arterial patency rates and reperfusion arryhtmias.

 In the primary PCI group, TIMI 3 patency was obtained in 83.3% of cases. There was no statistically significant relation between the presence of reperfusion arryhtmia and TIMI flow grades in primary PCI group although reperfusion arryhtmia was observed with a ratio of 82.2% in these patients with TIMI 3 flow.

42.3% and 38.1% of the patients who had received thrombolytic therapy had TIMI 3 and TIMI 2 flow, respectively. The incidence of reperfusion arryhthmia observed in the group of patients with TIMI 1 flow was significantly lower in thrombolytic therapy arm. On the other hand, there was no relationship between the presence of reperfusion arryhthmia and higher TIMI flow grades (TIMI 2 and TIMI 3) in the same group.

Wehrens et al. [11] compared the electrocardiographic changes following reperfusion in AMI patients to whom thrombolytic therapy, primary PTCA, or rescue PTCA were performed. The researchers demonstrated that electrocardiographic changes as a noninvasive tool associated with reperfusion did not provide sufficient information to clinicians to distinguish TIMI 2 and 3 flows from each other after thrombolytic therapy. This result is compatible with our results. Gressin et al., investigated the relationship between ventricular arrhytmias and arterial patency by recording ventricular arrhytmias in the first 24 hours and examining the angiographic views in a group of patients with acute myocardial infarction treated with thrombolytic therapy [12]. On the basis of ventricular arrhythmias detected; arryhtmia rates, in patients with TIMI 2 and 3 flow grades and in those without arterial patency (TIMI 0 or 1 flow grades), was found to be similar. These results suggest that some arrythmias may be due to different reasons such as ongoing ischemia or metabolic abnormalities. However, in another study, rates of sustained VT or AIVR occurring in the first 6 hours were found significantly higher in patients with arterial patency and these arrhytmias were defined as noninvasive indicators of early coronary reperfusion [13]. But it must be emphasized that the results and assessment of this study were limited to the detection of arrythmias in the first 6 hours, which is different from previous studies. 

Engelen et al. demonstrated that at least 97% of patients developed one ventricular reperfusion arrhythmia in a study group of 62 patients with acute anterior myocardial infarction who underwent primary PCI. Nonsustained VT, AIVR, and frequent VEA were the most frequently arrhythmias (similar to our findings) and ventricular arrhytmias were correlated with reduced and worsening left ventricular functions after the acute phase of myocardial infarction among this group of patients treated via primary PCI and concluded that reperfusion arrhythmias are noninvasive indicators of myocardial cell damage [14].

### 4.1. Study Limitations

The main limitation of the present study resides in its retrospective design. Larger scale studies may provide additional information for clinical applications.

## 5. Conclusion 

In conclusion, reperfusion arrhytmia frequency is not significantly different between patients treated by primary PCI and thrombolytics although PCI is known to provide better arterial patency. AIVR, which is so far known as a reperfusion arrhytmia, may indicate myocardial damage and ongoing ischemia rather than vascular patency. Further larger prospective studies are required to validate this hypothesis.

## Figures and Tables

**Table 1:** Demographic and clinical characteristics of patients.

	Treatment		*P*
	Primary PCI (*n* = 54)	Thrombolytic (*n* = 97)
	Mean ± SD (median)	Mean ± SD (median)
Age (year)	58.81 ± 13.66	58.41 ± 12.37	**0.854**
Male gender	41 (75.9%)	77 (79.4%)	**0.622**
Height (cm)	168.56 ± 7.66	169.24 ± 6.56	**0.566**
Weight (kg)	76.61 ± 13.37	77.65 ± 14.65	**0.668**
BMI (kg/m^2^)	26.82 ± 4.12	28.53 ± 15.08	**0.416**
CAD	83.35 ± 20.56	82.79 ± 21.62	**0.877**
SBP (mmHg)	134.19 ± 25.13	139.28 ± 31.14	**0.305**
EF (%)	55.70 ± 10.15	54.24 ± 9.64	**0.381**
Time to revascularization (hours)*	4.25 ± 3.83	3.35 ± 1.87	**0.317**
HT	29 (53.7%)	48 (49.5%)	**0.619**
DM	14 (25.9%)	15 (15.5%)	**0.118**
Smoking	26 (48.1%)	39 (40.2%)	**0.345**
Previous MI	3 (5.6%)	9 (9.3%)	**0.418**
Preinfarction angina	37 (68.5%)	52 (53.6%)	**0.074**
In hospital CV event	4 (7.4%)	5 (5.2%)	**0.575**

PCI: percutaneous coronary intervention, *n*: number, SD: standard deviation, BMI: body mass index, CAD: coronary artery disease, SBP: systolic blood pressure, EF: left ventricular ejection fraction, HT: hypertension, DM: diabetes mellitus, MI: myocardial infarction, and CV: cardiovascular.

Student *t*-test and *Mann Whitney *U*-tests were used for comparison of the groups.

**Table 2:** Laboratory measures of patients.

	Treatment		*P*
	Primary PCI (*n* = 54)	Thrombolytic (*n* = 97)
	Mean ± SD (median)	Mean ± SD (median)
Glucose (mg/dL)	184.28 ± 88.99 (154.50)	168.80 ± 76.74 (143)	**0.390**
Urea (mg/dL)	37.51 ± 16.36	37.77 ± 12.01	**0.913**
Potassium (mg/dL)	4.25 ± 0.73	4.39 ± 0.61	**0.228**
Creatinine (mg/dL)*	0.99 ± 0.23 (1)	1.22 ± 1.36 (1)	**0.191**
Troponin I (ng/mL)*	4.62 ± 11.19 (0.09)	2.52 ± 9.08 (0.07)	**0.314**
HDL-C (mg/dL)	36.30 ± 10.33	37.97 ± 9.76	**0.324**
LDL-C (mg/dL)	113.03 ± 33.53	117.67 ± 39.15	**0.464**
Total C (mg/dL)	186.54 ± 53.53	189.53 ± 49.46	**0.730**
Triglyceride (mg/dL)*	177.09 ± 238.81 (113)	165.34 ± 126.47 (119)	**0.307**

PCI: percutaneous coronary intervention, *n*: number, SD: standard deviation, C: cholesterol, HDL-C: high density lipoprotein cholesterol, and LDL-C: low density lipoprotein cholesterol.

Student *t*-test and *Mann Whitney *U*-tests were used for comparison of the groups.

**Table 3:** Comparison of the frequency of various reperfusion arrythmias between primary percutaneous coronary intervention and thrombolytic treatment groups of patients.

	Treatment		*P*
	Primary PCI (*n* = 54)	Thrombolytic (*n* = 97)
	Mean ± SD (median)	Mean ± SD (median)
	*n* (%)	*n* (%)
Reperfusion arrythmias*	45 (83.3%)	86 (88.7%)	**0.355**
AIVR	27 (50%)	71 (73.2%)	**0.004**
Sustained VT	4 (7.4%)	5 (5.2%)	**0.575**
Nonsustained VT	31 (57.5%)	68 (70.1%)	**0.116**
Ventricular fibrillation**	2 (3.7%)	0 (0%)	**0.126**
AV Block	3 (5.6%)	6 (6.2%)	**0.875**
Frequent PVCs	5 (9.3%)	17 (17.5%)	**0.168**
Atrial fibrillation	7 (13%)	26 (26.8%)	**0.049**

PCI: percutaneous coronary intervention, *n*: number of patients, SD: standard deviation, AIVR: accelerated idioventricular rhythm, VT: ventricular tachycardia, AV: atrioventricular, and PVCs: prematüre ventricular contractions.

*Detected in the first 48 hours.

Chi-square test and **Fisher's Exact Chi-square tests were performed to analyse data.

**Table 4:** Comparison of electrocardiographic ST segment regression, TIMI flow grades, and the presence of collateral circulation between two treatment groups.

		Treatment		*P*
		Primary PCI (*n* = 54)	Thrombolytic (*n* = 97)
		*n* (%)	*n* (%)
ST segment regression	<50%	11 (20.4%)	50 (51.5%)	0.001*
	>50%	43 (79.6%)	47 (48.5%)	

TIMI flow grade	TIMI 0	0 (0%)	7 (7.2%)	**0.050**
	TIMI 1	3 (5.6%)	12 (12.4%)	**0.258**
	TIMI 2	6 (11.1%)	37 (38.1%)	0.001*
	TIMI 3	45 (83.3%)	41 (42.3%)	0.001*

Presence of collateral vessels		8 (14.8%)	25 (25.8%)	**0.118**

TIMI: Thrombolysis In Acute Myocardial Infarction, PCI: percutaneous coronary intervention, and *n*: number of patients.

Chi-square test and Fisher's Exact Chi-square tests were performed to analyse data.

*Values of *P* < 0.05 indicate statistically significant data.

**Table 5:** Association between the occurrence of reperfusion arrythmias and ST segment regression in primary PCI and thrombolytic treatment groups.

Treatment	ST segment regression	Reperfusion arrythmias (in the first 48 hours)		*P*
		Present	Absent
		*n* (%)	*n* (%)
Primary PCI	<50%	10 (22.2%)	1 (11.1%)	**0.450**
	>50%	35 (77.8%)	8 (88.9%)	

Thrombolytic	<50%	40 (46.5%)	10 (90.9%)	0.006*
	>50%	46 (53.5%)	1 (9.1%)	

PCI: percutaneous coronary intervention, *n*: number of patients.

Chi-square test was performed to analyse data. *Values of *P* < 0.05 indicate statistically significant data.

**Table 6:** The relationship between presence of reperfusion arrythmias and TIMI flow grades in different treatment groups.

Treatment	TIMI flow grade	Reperfusion arrythmias (in the first 48 hours)		*P*
		Present	Absent
		*n* (%)	*n* (%)
Primary PCI	TIMI 1	2 (4.4%)	1 (11.1%)	**0.428**
	TIMI 2	6 (13.3%)	0 (0%)	**0.245**
	TIMI 3	37 (82.2%)	8 (88.9%)	**0.624**

Thrombolytic	TIMI 0	7 (8.1%)	0 (0%)	**0.326**
	TIMI 1	8 (9.3%)	4 (36.4%)	0.010*
	TIMI 2	33 (38.4%)	4 (36.4%)	**0.897**
	TIMI 3	38 (44.2%)	3 (27.3%)	**0.285**

TIMI: Thrombolysis In Acute Myocardial Infarction, PCI: percutaneous coronary intervention, and *n*: number of patients. Chi-square test and Fisher's Exact Chi-square tests were performed to analyse data.

*Values of *P* < 0.05 indicate statistically significant data.

**Table 7:** Infarct related artery and presence of reperfusion arrythmias in both treatment groups.

Treatment	IRA	Reperfusion arrythmias (in the first 48 hours)		*P*
		Present	Absent
		*n* (%)	*n* (%)
Primary PCI	LAD	28 (62.2%)	2 (22.2%)	0.027*
	RCA proksimal	3 (28.9%)	6 (66.7%)	0.001*
	CX	4 (8.9%)	1 (11.1%)	**1.000**

Thrombolytic	LAD	44 (51.2%)	4 (36.4%)	**0.355**
	RCA proksimal	33 (38.4%)	7 (63.6%)	**0.109**
	CX	9 (10.5%)	0 (0%)	**0.260**

IRA: Infarct related artery, PCI: percutaneous coronary intervention, LAD: left anterior descending artery, RCA: right coronary artery, and CX: circumflex artery.

Chi-square test and Fisher's Exact Chi-square tests were performed to analyse data.

*Values of *P* < 0.05 indicate statistically significant data.
